# Transcutaneous electrical nerve inhibition using medium frequency alternating current

**DOI:** 10.1038/s41598-022-18974-3

**Published:** 2022-09-01

**Authors:** Seppe Maris, Michiel Brands, Daniele Lenskens, Geert Braeken, Stefan Kemnitz, Herbert Vanhove, Myles Mc Laughlin, Raf Meesen, Bert Brône, Björn Stessel

**Affiliations:** 1 Department of Anaesthesiology and Pain Medicine, Jessa Hospital, Stadsomvaart 11, 3500 Hasselt, Belgium; 2 Faculty of Medicine and Life Sciences, BIOMED Research Institute, UHasselt, Agoralaan Gebouw C, 3590 Diepenbeek, Belgium; 3 Department of Anaesthesiology and Pain Medicine, University Hospital Leuven, Herestraat 49, 3000 Leuven, Belgium; 4 Exp ORL, Department of Neurosciences, The Leuven Brain Institute, KU Leuven, Herestraat 49, 3000 Leuven, Belgium; 5 Neuroplasticity and Movement Control Research Group, Rehabilitation Research Institute (REVAL), Hasselt University, Agoralaan Gebouw A, 3590 Diepenbeek, Belgium; 6 Movement Control and Neuroplasticity Research Group, Group Biomedical Sciences, Department of Movement Sciences, KU Leuven, Herestraat 49, 3500 Leuven, Belgium

**Keywords:** Nervous system, Quality of life

## Abstract

Transcutaneous medium-frequency alternating electrical current is defined as an alternating current between 1 and 10 kHz and is capable of producing an instant, reversible block. This study aims to evaluate the efficacy of sensory perception and force production of the index and middle finger after transcutaneous medium-frequency alternating electrical current stimulation of the distal median nerve. A single-center prospective interventional cohort study was conducted in adult healthy volunteers at the Jessa Hospital, Hasselt, Belgium. Two different electrodes (PALS & 3M) were placed on the distal median nerve, which was located using a Sonosite X-Porte Ultrasound transducer, with the first electrode being placed on the skin at the level of the transverse carpal ligament and the second electrode 7 cm proximally to the first electrode. The tactile sensation was evaluated with Semmes–Weinstein monofilament test and sensation of pressure/pain was evaluated with an algometer. Peak force production was assessed with an electronic dynamometer. All measurements were performed at baseline and tMFAEC stimulation frequencies of 2 and 10 kHz in a randomized manner. Statistical analysis was performed with a one-way ANOVA with repeated measures test or a Friedman rank sum test, followed by the Wilcoxon signed rank test adjusted with Bonferroni correction. A *p*-value < 0.05 was considered statistically significant. From 9 to 13th of April 2021, 25 healthy volunteers were included in the Jessa Hospital, Hasselt, Belgium. A statistically significant reduction in tactile sensation during 2 kHz and 10 kHz stimulation compared to baseline was observed (2.89 ± 0.22 (PALS2); 3.35 ± 0.25 (3M2) and 2.14 ± 0.12 (PALS10); 2.38 ± 0.12 (3M10) versus − 1.75 ± 0.09 (baseline), *p* < 0.0001). 3M electrodes showed a tendency towards the elevation of pressure pain threshold compared to baseline. No significant difference in mean peak forces of the index and middle fingers after transcutaneous medium-frequency alternating electrical current stimulation with 2 and 10 kHz was found. This study demonstrates that transcutaneous medium-frequency alternating electrical current stimulation on the distal median nerve inhibits tactile sensory nerve activity in the index and middle finger when stimulation of 2 kHz and, to a lesser extent, 10 kHz was applied. A reduction of motor nerve activity was not observed but force production measurements may be prone to error.

Trial registration: clinicaltrials.gov on 01/04/2021. NCT-Number: NCT04827173.

## Introduction

Orthopedic surgery is traditionally performed under general or locoregional anesthesia. These anesthetic techniques may be accompanied by postoperative nausea or prolonged numbness, respectively, which may be perceived as uncomfortable for the patient^1^. General and locoregional anesthesia inevitably carries risks regarding patient safety and is a time-consuming procedure. In the case of orthopedic surgical procedures like minimal-invasive hand surgery, it might be useful to develop a rapid onset anesthetic technique without long-lasting patient-related side effects.

The mechanism of action potential generation using electrical currents, such as transcutaneous electrical nerve stimulation and neuromuscular electrical stimulation, is a widely adopted technique in the field of chronic pain management and rehabilitation^2–5^. Using transcutaneous medium-frequency alternating electrical current (tMFAEC), this study aimed to investigate an experimental technique that might eliminate the risk of unwanted side-effects associated with general or locoregional anesthesia.

tMFAEC is defined as an alternating current with a frequency in the range of 1–10 (kHz) and has unique nerve blocking properties that can produce a very localized nerve inhibition depending on the placement of the electrodes^6^. A minimum amplitude is mandatory to achieve a nerve block, this is called the block threshold^7^. Although it is important to note that the block threshold will also depend on frequency, pulse width and waveform. When this threshold is exceeded, the block arises nearly instant and is rapidly reversible when the stimulus is turned off^8,9^. The nerve responses produced are dependent on the frequency and amplitude of the waveform and the duration of the stimulus^9^. The application of a tMFAEC impulse that is too short will not result in a nerve block.

The continuous use of very high amplitudes and frequencies may lead to prolonged numbness or even irreversible damage to the nerve^9,10^.

This study is based on research by Kim et al., which focused on future implications of tMFAEC for rehabilitation purposes in combination with existing rehabilitation devices, such as robotic exoskeletons to inhibit unwanted sensorimotor activity^11^. In this study with a small sample size of 8 healthy volunteers, tMFAEC stimulation at 10 kHz was applied to the median nerve by placing electrodes on both the skin overlying the median nerve near the transverse carpal ligament and the ipsilateral olecranon process. Latter localization is unexpected since the median nerve is not located near the ipsilateral olecranon process. Albeit, Kim et al. concluded that tMFAEC stimulation at 10 kHz immediately inhibits both sensory and motor nerve activity. Furthermore, motor forces recovered immediately after stimulation cessation. More specifically, at maximum tMFAEC stimulation intensity (mean 31.4 mA), the pain thresholds increased 43% and finger forces were reduced by approximately 40%^11^.

The present study focused on the potential anesthetic effects of tMFAEC stimulation. More specifically, it concentrated on whether tMFAEC can be implemented as a safe and effective new anesthetic technique for distal limb surgery or at least as a rescue anesthetic strategy in case of a failed locoregional anesthetic blockade. It was clear that the results of Kim et al. are encouraging but that pain threshold increase should be further promoted by the implementation of intervention adjustments.

The main aim of the present study was to confirm the results of Kim et al., considering the small sample size of this study and the odd location of the proximally placed electrodes. Therefore, the present study aimed to assess the anesthetic efficacy and safety of tMFAEC stimulation at 10 kHz in a larger sample of 25 healthy volunteers. Ultrasound guidance was used to determine localization and depth of the median nerve in an attempt to improve electrode placement. We hypothesized that tMFAEC stimulation at 10 kHz on the distal median nerve would reduce the sensory perception and inhibit force production in the index and middle finger to the same extent or even to a greater degree compared to Kim et al. considering the improved electrode placement. A second key objective was to assess the efficacy of tMFAEC stimulation at 2 kHz in producing anesthesia and to compare these results with the results of tMFAEC stimulation at 10 kHz.

## Materials and methods

### Study design

In this single-center prospective interventional feasibility study, the effect of tMFAEC stimulation on sensory and motor inhibition was evaluated in 25 healthy volunteers.

This study was conducted on healthy volunteers older than 18 years of age. Participants were excluded if they had a previous medical history of musculoskeletal pain, a neurologic disorder, diabetes mellitus, hypertension, an autoimmune disease, or any type of surgical history in their right arm. Furthermore, subjects who showed an allergic reaction to the stimulation electrodes were also excluded. After obtaining written informed consent, 25 participants were included for this study between the 9 to 13th of April 2021.

In this study, the subjects acted as their own controls since baseline measurements with 0% tMFAEC stimulation were compared to the measurements performed at 100% tMFAEC stimulation intensity.

### Randomization & blinding

All subjects underwent an identical experimental setup and testing conditions. All participants were blinded for the applied stimulation frequency and electrode to reduce the possibility of anticipation. The order of testing different frequencies and electrodes was randomly allocated using the Random Allocation Software developed by Saghaei, M^12^.

### Outcome measures

The primary outcome was defined as the assessment of peripheral sensory perception and force production in the index and middle finger when tMFAEC was applied at a frequency of 10 kHz to the distal median nerve.

The key secondary outcome was the assessment of peripheral sensory perception and force production in the index and middle finger with tMFAEC at a frequency of 2 kHz.

Other secondary outcome parameters were confirmation of the participant’s safety, the effect of the use of two different types of electrodes, and the effect of differences in depth of the median nerve at mid-forearm level on the anesthetic efficacy of the nerve block.

The effect of a different frequency (i.e. 2 kHz) on the level of sensorimotor inhibition was evaluated during the same experiment. For this frequency, the experimental setup was identical to the 10 kHz stimulation. Furthermore, the effect of two substantially different electrodes was evaluated. The surface electrodes used were PALS Platinum Electrodes 32 × 32 mm (PALS Electronics Ltd., Dudullu OSB, Istanbul, Turkey) and 3M Red Dot Elektrode, 2228 18 × 18 mm (3M, Diegem, Belgium). Supplementary fig. S1 gives an overview of the two electrodes’ shape and size.

Patient safety was assessed by evaluating the sensory and motor function of the median nerve after completion of the study. To maximize patient safety, the maximum current intensity tolerable was determined for every participant. This was assessed by gradually increasing tMFAEC stimulation to individual pain threshold with a current intensity limit of 35 mA.

### Study procedures

**Figure 1:**
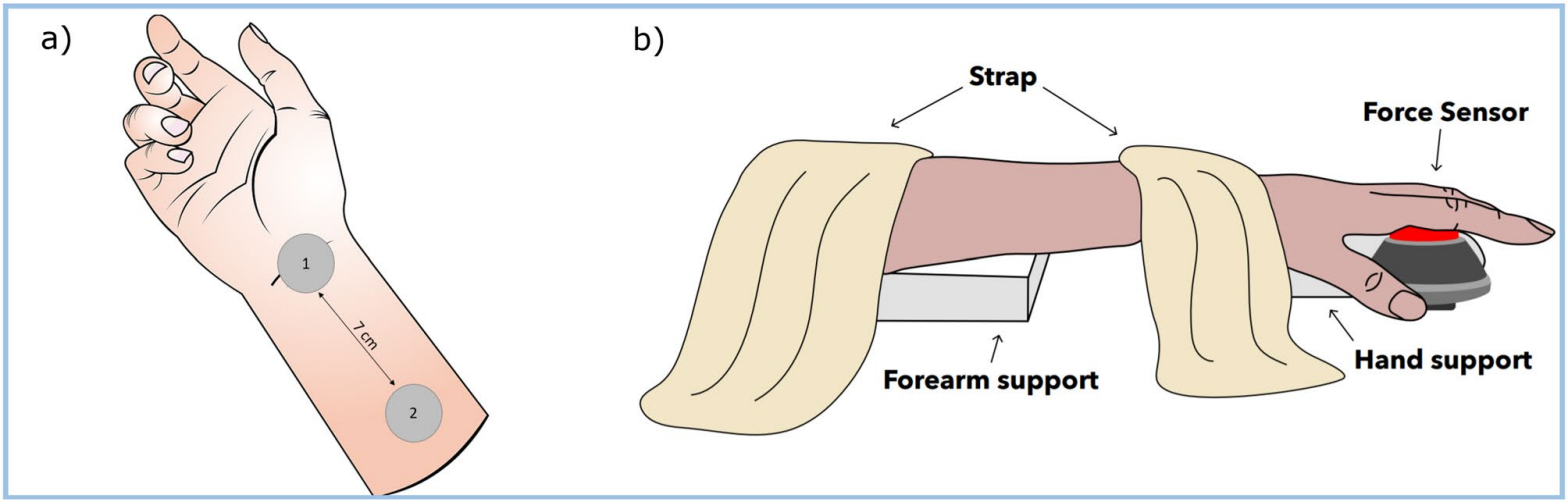
(**a**) Placement of the electrodes. Two electrodes are placed on the lower arm of the subject. Electrode 1 is placed on top of the carpal ligament. Electrode 2 is placed seven cm superior to electrode 1. (**b**) Fixation of the forearm. A forearm support and two straps keep the arm in a fixed position to prevent external forces to influence the force production assessment of the index and middle finger.

The localization and depth of the right median nerve were assessed using a Sonosite X-porte ultrasound machine with a high-frequency linear transducer HFL38 (15–6 MHz) (FUJIFILM Sonosite, Inc.; Bothwell, WA, USA). The depth was reported in millimeters on two locations of the right forearm: at the level of the transverse carpal ligament and 7 cm more proximal. The skin covering the median nerve was cleaned with ether to remove superficial lipids and decrease skin impedance^13^.The two electrodes were placed on the two locations as mentioned above (Fig. 1a).

Electrode placement was slightly adjusted in a more lateral direction if tMFAEC stimulation induced undesired muscle contraction secondary to direct stimulation of the neuromuscular junction or stimulation of the ulnar nerve. Subsequently, the participant was seated on a chair and the right arm was placed on an armrest in a neutral resting position.

First, baseline measurements without tMFAEC stimulation were performed. Electrodes were applied on the participant's forearm and no current was generated. When baseline data were collected, measurements of tMFAEC stimulation at 2 kHz and 10 kHz were performed in a randomized order.

tMFAEC stimulation was generated with a DS5 Isolated Bipolar Constant Current Stimulator (Digitimeter, Hertfordshire, UK), coupled to a driver (National Instruments Corp., Austin, TX, USA). To create a biphasic, steady, unmodulated alternating current of 10 kHz or 2 kHz, a sinusoidal waveform was generated using a signal generator (NI USB-6216, National Instruments, Austin, TX) and this was delivered to the DS5 Isolated Bipolar Constant Current Stimulator (Digitimer, Hertfordshire, UK) input. The DS5 has a maximum output current of 50 mA, a compliance voltage of 160 V and a bandwidth of 20 kHz. Custom software written in Matlab (Mathworks, Natick, MA, USA) was used to control the signal generator and thus deliver the desired waveforms to the DS5 current source. Before the experiment, all waveforms were delivered over a 3 kHOhm resistor and visualized on an oscilloscope to ensure that the correct waveforms and currents were delivered. Additionally, the DS5 has a screen that shows the current, voltage, and impedance during stimulation. If the compliance voltage is reached, i.e. the DS5 is not able to deliver the requested current, an audio alarm sounds. During the testing of healthy volunteers, the experimenter monitored the information to ensure that all stimulation was within the compliance limits of the DS5.

Before testing, the intolerance threshold for all four possible combinations of tMFAEC stimulation frequency and electrode type was examined for every participant. Stimulation with alternating current started at a very low intensity and was gradually increased in augmenting steps of 2 mA until the participant reported significant discomfort. This detected threshold intensity, reported in mA, was used to perform the tests for that given combination. To additionally ensure patient safety, NRS pain scores were registered for every condition.

#### Tactile sensation

Testing of tactile sensation was performed using the Semmes–Weinstein monofilament examination. A set of 20 nylon monofilaments (Sammons Preston Rolyan, Cedarburg, WI, USA), graded according to monofilament diameter, was used. Each monofilament was applied to the skin of the index finger, beginning with the smallest diameter to obtain an ascending method of threshold testing. All monofilaments were held in contact with the skin until they bent (creating the form of the letter ‘C’) and were removed after 1 s. During this test, the participant was blinded and had to indicate if the sensation of the monofilament was present. For the 2 kHz and 10 kHz measurements, tMFAEC stimulation was administered for 5 s followed by monofilament application. This method eliminated the anticipation of the participant for the onset of the monofilament stimulation. Tactile threshold was recorded in gram (g) force, as directed by the manufacturer and the force values were presented on a logarithmic scale.

#### Pressure pain threshold

To evaluate pressure sensation or pressure pain, a 1-cm Wagner FDX algometer (Wagner Instruments, Greenwich, CT, USA) was used. The pressure was applied with the algometer in a perpendicular direction on the thenar musculature of the right thumb. The participants were instructed to indicate when they felt a transition from pressure to pain, corresponding to each participant’s pressure pain threshold. The pressure was increased gradually and released as soon as the subject reported pain. tMFAEC stimulation was also discontinued after the subject’s pressure pain threshold was reached. Threshold values for pressure pain were recorded in kilogram (kg) force.

#### Force production

To test the effect of tMFAEC stimulation on force production, a peak force production assessment was performed. An arm support was placed underneath the arm and wrist to maintain approximately 0° wrist extension and metacarpophalangeal flexion. After that, two straps were used to fix the participant’s wrist and forearm to a testing platform to limit force transmission from proximal muscles or elbow and shoulder joints (Fig. 1b). The participants were asked to press a force sensor, i.e. an electronic handheld dynamometer: CompuFET2 (ProCare, Groningen, Netherlands).

The wrist and hand positions were optimized to maximize the contribution of the intrinsic hand muscles. Subjects were asked to press the sensor using their maximum voluntary contraction (MVC) to determine the target force. A digital monitor provided visual feedback on index and middle finger peak forces during the MVC measurements. The MVC measurement was repeated 3 times and the average values were used for analysis. The virtual finger is defined as the mean force production of the index and middle finger force.

All experiments were repeated under 5 different conditions: baseline conditions, with PALS electrodes at 10 kHz and 2 kHz and with 3M Red Dot electrodes at 10 kHz and 2 kHz. Following every experiment, a recovery period of one minute was allowed to the participant to mitigate the effects of neurotransmitter depletion on nerve conduction.

### Statistical analysis

For a feasibility study, a formal sample size calculation may not be appropriate but a sample size justification is necessary^14^. Compared to Lee et al., we tripled the sample size and included 25 participants. This sample size should allow to estimate the standard deviation of sensory nerve inhibition which can be used in a sample size calculation for a full-scale trial^15^.

Statistical analysis was performed using IBM SPSS v.26.0 for Windows. (IBM, New York, NY, USA). Data were analyzed for normal distribution with the Shapiro–Wilk test. If data were distributed normally, values were presented as mean ± standard error of the mean (SEM). If data were not normally distributed, values were presented as median with interquartile range (IQR), and non-parametric tests were performed to test for significance.

Confirmation of participant’s safety of tMFAEC by determining the maximum current intensity tolerable to a participant was presented with descriptive statistics and a Friedman test. Comparison of the electrodes and frequencies for pain thresholds was performed using a Wilcoxon Signed Rank test. Evaluation of the effect of two different electrodes with substantially different materials on the level of sensorimotor inhibition was presented using descriptive statistics.

To compare the tactile threshold on the level of sensory inhibition between baseline and tMFAEC stimulation with two different stimulation frequencies i.e. 2 kHz and 10 kHz a one-way ANOVA with repeated measures was used.

A Friedman test was used to compare pressure pain thresholds among baseline and 100% intensity of tMFAEC stimulation in the thenar musculature of the right thumb. To analyze the exact significant differences, a Wilcoxon Signed Rank test was performed.

To compare peak index and middle finger forces using normalized mean values, a one-way ANOVA with repeated measures was used. To test for significance, a Bonferroni correction to the ANOVA test was applied.

To test for a possible association between the depth of the median nerve and the effect of tMFAEC, a Spearman rank test was performed.

A *p*-value < 0.05 was considered statistically significant, while *p* < 0.10 was considered a tendency.

### Ethics approval and consent to participate

This study was approved by the ethical committee of the Jessa Hospital, Hasselt, Belgium (Chairperson Dr. Koen Magerman) on March 12th, 2021, registered on clinicaltrials.gov on 01/04/2021 (NCT04827173), and executed according to the Declaration of Helsinki. Patients were able to participate after receiving oral and written information about the study and after signing an informed consent form.

## Results

**Figure 2:**
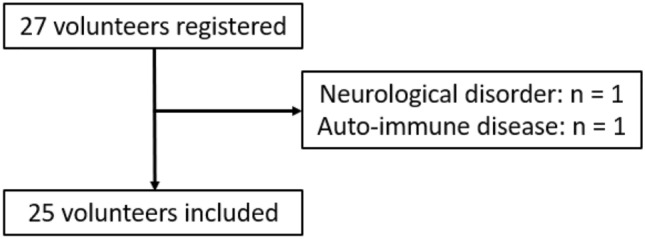
Flow chart of patient selection and exclusion.

A flow chart of patient selection and exclusion is presented in Fig. 2. Baseline characteristics of the participants are presented in Table 1.

**Table 1:** Characteristics of the study population.

Age (years)	33.12 ± 2.66
Gender (male, %)	11 (44.0%)
BMI (kg/cm^2^)	23.7 (21.0–26.4)
Dominant hand (right, %)	21 (84.0%)
Depth *median nerve* at transverse carpal ligament (mm)	4.68 ± 0.33
Depth *median nerve* 7 cm superior to transverse carpal ligament (mm)	12.44 ± 0.46

Data represented as mean ± SEM. Gender and Dominant hand are represented as percentages (%). BMI is represented as median (IQR). *BMI* Body Mass Index.

tMFAEC stimulation resulted in an unpleasant burning or tingling sensation in the sensory supply area of the median nerve. The sensation was not experienced as painful, as can be deducted from the NRS pain scores in supplementary table S1. The sensation was directly proportional to tMFAEC stimulation intensity and resulted in an intolerance threshold intensity. The detected intolerance threshold intensities for all four possible combinations of tMFAEC stimulation frequency and electrode type are presented in Table 2. The intolerance threshold intensity at 10 kHz stimulation was significantly higher compared to the threshold at 2 kHz stimulation (Wilcoxon Signed Rank Test, *p* < 0.001). The intolerance threshold for the PALS electrodes was significantly higher compared to the 3M Red Dot electrodes (Wilcoxon Signed Rank Test, *p* < 0.01).

**Table 2:** Intolerance threshold intensities. Data represented as median (IQR) for pain thresholds.

Intolerance threshold PALS 10 kHz (mA)	34.0 (21.0–35.0)
Intolerance threshold PALS 2 kHz (mA)	10.0 (8.0–13.0)
Intolerance threshold 3M Red Dot 10 kHz (mA)	23.0 (17.0–30.0)
Intolerance threshold 3M Red Dot 2 kHz (mA)	8.0 (6.0–9.0)

**Figure 3:**
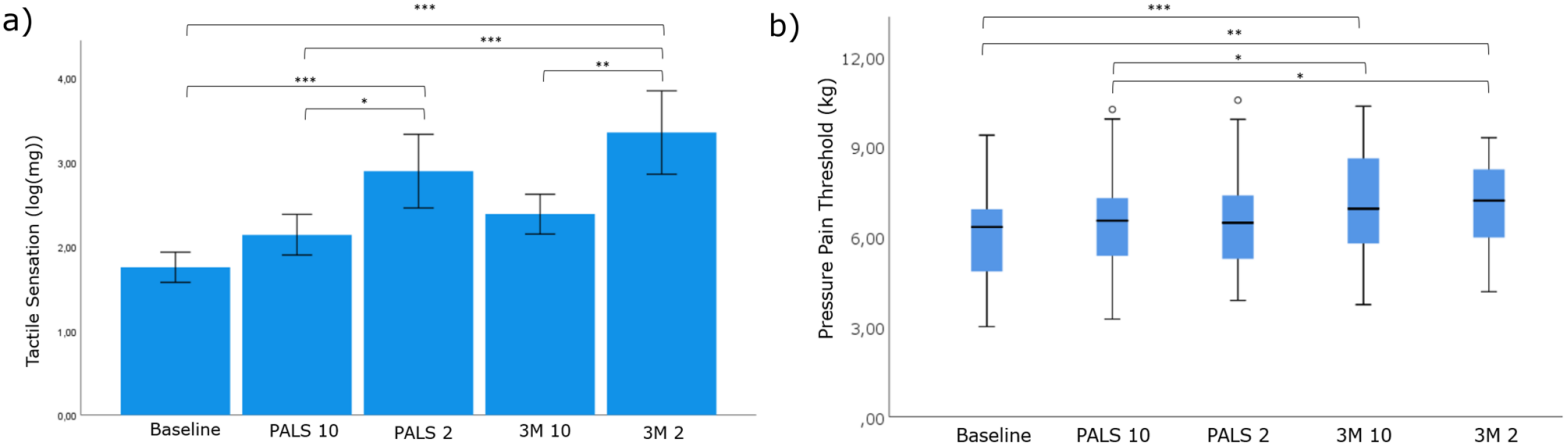
(**a**) Mean tactile sensation with the Semmes–Weinstein Monofilament test. Values for tactile sensation were transformed from a logarithmic scale to a linear scale to perform analysis. *p < 0.05; **p < 0.01; ***p < 0.001, one-way ANOVA with Bonferroni adjustment. (**b**) Pressure pain threshold test with the pressure algometer. Values for pressure pain threshold are presented in a boxplot. *p < 0.05; **p < 0.01; ***p < 0.001, Wilcoxon Signed Rank test.

Figure 3a shows the results of the testing of tactile sensation with the Semmes–Weinstein Monofilament Test. Significant results were found between baseline and the four possible intervention combinations (one-way ANOVA, *p* < 0.001). Sensory inhibition at 2 kHz stimulation frequency is significantly higher compared to baseline for both PALS and 3M Red Dot electrodes (one-way ANOVA with Bonferroni adjustment: *p* < 0,001). Stimulation with PALS electrodes and 3M electrodes at 2 kHz resulted in a consistently stronger block of tactile sensation compared to stimulation at 10 kHz (one-way ANOVA with Bonferroni adjustment: *p* = 0.022).

A Friedman test shows a borderline significant difference in pressure pain thresholds between the groups (*p* = 0.050).When groups are tested pairwise with a Wilcoxon Signed Rank Test, statistically significant differences can be found. (Fig. 3b). The most significant elevation of pressure pain threshold was noticed with 3M Red Dot electrodes compared to baseline.

**Figure 4:**
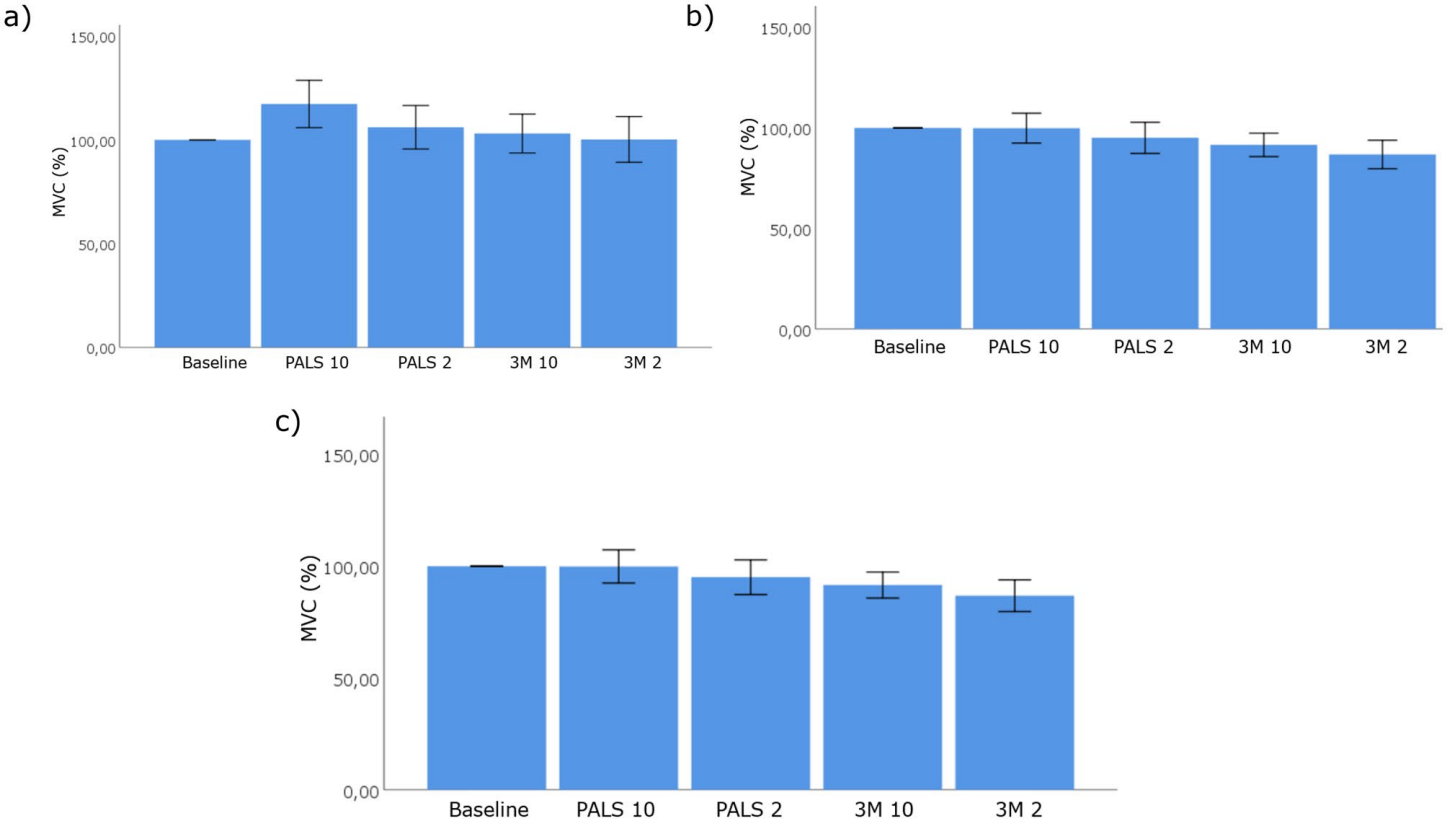
Mean peak forces of the index (**a**) and middle finger (**b**) for the 5 different conditions. The mean value of the index and middle finger forces results in virtual finger forces (**c**). Data represented as mean ± SEM, normalized by baseline MVC. No significant differences in peak finger forces have been found with one-way ANOVA with Bonferroni adjustment. MVC, Maximum Voluntary Contraction.

Figure 4a,b show the normalized mean MVC for the index finger and middle finger respectively. Figure 4c shows the normalized mean virtual finger MVC. A one-way ANOVA with Bonferroni adjustment shows no significant effect of tMFAEC stimulation on the MVC within the different conditions. One patient suffered from a tetanic contraction during tMFAEC stimulation. Supplementary table S2 shows the exact values for the mean tactile sensation, median pressure pain, and mean MVC experiments.

Table 3 presents the results of the Spearman Rank test, which shows no overall correlation between the depth of the median nerve and the effect of tMFAEC.

**Table 3:** Association between depth of *median nerve* and effect on different test outcomes.

	Condition	Depth 1	*p*-value	Depth 2	*p*-value
Tactile sensation	PALS 10	0.074	0.726	− 0.246	0.237
	PALS 2	− 0.133	0.525	− 0.295	0.152
	3M 10	− 0.489	**0.013**	− 0.468	**0.018**
	3M 2	− 0.305	0.138	− 0.299	0.147
Pressure pain	PALS 10	− 0.350	0.867	− 0.140	0.503
	PALS 2	− 0.103	0.624	0.472	0.472
	3M 10	− 0.041	0.845	− 0.122	0.561
	3M 2	0.271	0.271	− 0.263	0.204
MVC Virtual	PALS 10	0.369	0.359	0.100	0.635
	PALS 2	0.383	0.059	0.280	0.280
	3M 10	0.438	**0.029**	0.085	0.687
	3M 2	0.373	0.066	0.043	0.837

Testing for a significant association is done with the Spearman Rank test. Results are given as correlation coefficients.

Significant *p*-values are in bold.

## Discussion

There is a wide body of evidence in the application of low frequency and medium frequency currents in the management of acute and chronic pain states. In contrast, only a few small studies investigate pain modulation using medium-frequency alternating current stimulations (1–20 kHz)^11,16–18^.

The present study results suggest that tMFAEC stimulation inhibits sensory nerve activity at the level of the distal forearm without inhibiting motor nerve activity, both at 2 kHz and 10 kHz. More specifically, the deepest block of tactile sensation was achieved with maximal tolerable tMFAEC stimulation at 2 kHz using 3M electrodes. The most significant elevation of pressure pain thresholds were noted with 3M electrodes at both 2 kHz and 10 kHz. The observed inhibition of tactile sensation was much more profound compared to the observed inhibition of pressure pain. A correlation between the depth of the median nerve and the effect of tMFAEC stimulation on nerve activity could not be detected. These results are not consistent with the findings of Kim et. al. They concluded that tMFAEC stimulation at 10 kHz inhibits both sensory and motor nerve activity^11^. Furthermore, the described level of tactile and pressure pain inhibition with tMFAEC stimulation at 10 kHz in latter study was more profound than the observed inhibition in this study. In contrast to our results, they also observed that the level of inhibition of tactile sensation and pressure pain was in the same range. Noteworthy, pressure pain threshold at baseline of latter study was less than half compared to the present study.

These conflicting results may be explained by a set of factors. First, we chose to alter the location of the proximally placed electrode from the ipsilateral olecranon process to an ultrasound-guided placement at the mid-forearm level. Hypothetically, motor branches of the median nerve responsible for innervation of the lumbrical muscles of index and middle finger are already given off at that level. This hypothesis would explain the absence of motor nerve inhibition observed in this study. However, it is described that the palmar digital branch, responsible for innervation of the lumbrical muscles of index and middle finger terminates from the median nerve at carpal tunnel level. Another more plausible explanation for the absence of motor innervation inhibition would be that small differences in experimental setup would have allowed participants to apply high levels of pressure on the dynamometer without contribution of the intrinsic hand muscles.

Second, the discrepancy in observed level of tactile sensation inhibition and pressure pain inhibition might be explained by the fact that pressure pain threshold at baseline in this study was twice as high compared to Kim et al.^11^. A further increase in pressure applied with the algometer on the right thumb may have readily elicited a pain sensation in an area of the thumb innervated by the radial nerve. Third, the low pressure pain threshold described by Kim et al. can be explained by the inability to blind participants with risk of performance bias.

Interestingly, the results showed a significantly higher tactile sensation threshold for 2 kHz stimulation frequencies compared to 10 kHz stimulation frequencies. This indicates that at lower stimulation frequency, sensory inhibition is more effective. These effects appeared at a lower stimulus intensity (mA) compared to the 10 kHz group. These findings are remarkable and hard to explain. This would be an interesting topic for future research.

The effect of tMFAEC stimulation on nerve activity was also tested and analyzed with two different types of electrodes (PALS vs. 3M). 3M electrodes were clearly more effective in increasing tactile sensation threshold compared to PALS electrodes. When comparing groups pairwise to study pressure pain inhibition, a similar effect could be noticed. The overall better results with the 3M electrodes could be due to the smaller contact area of the 3M electrodes. A smaller contact area with the same current intensity results in a higher current density. Larger energy levels can be delivered through the electrodes to the median nerve and may result in a more profound nerve block. To test the isolated effect of the composition of the electrodes, electrodes with truly identical contact areas should be compared. PALS 32 × 32 mm electrodes were used in this study, but 3M Red Dot electrodes with an equal contact area are not available. Therefore, a correct interpretation of the effect of the electrode material composition is not possible.

Regarding patient safety and comfort, PALS electrodes were found to be more tolerable (at the level of the forearm) than the 3M electrodes resulting in a higher intolerance threshold intensity with PALS electrodes. This can be explained by the larger contact area of PALS electrodes. The unpleasant burning or tingling sensation was not experienced in the skin–electrode contact area but in the sensory supply area of the median nerve (skin over palmar side of the thumb, index, middle finger and part of the ring finger and thumb side of the palm). Additionally, our results showed that tMFAEC stimulation at 10 kHz frequency was also more tolerable compared to 2 kHz frequency resulting in a higher intolerance threshold intensity at 10 kHz. A small trial by Serrano-Munoz et al., which compared muscle strength inhibition between 5 and 10 kHz stimulation, observed a similar finding^19^. In our study, this finding impeded progressive augmentation of the stimulation intensity in the 2 kHz group to equivalent current intensity levels as in the 10 kHz groups. This observation needs to be taken into account in future study designs. If the anesthetic effect of tMFAEC stimulation is too low at the intolerance threshold of the patient, the clinical applicability is absent. No other harmful effects to the study participants were observed.

Finally, the effect of the depth of the median nerve on the level of sensorimotor inhibition was analyzed. No significant overall correlation was observed.

The strengths of the present study include the rather large sample size, the ultrasound guided placement of the electrodes and the combined testing at different frequencies with different types of electrodes.

This study also has some limitations. Firstly, pain is a personal sensation with a strong affective component and is prone to sensitization mechanisms. Variability in pain scoring was reduced as much as possible by randomization and blinding for testing frequencies. However, the intolerance threshold, in general, has a high inter-individual variation and is caused by multiple factors.

Blinding and randomization cannot eliminate all these variables, so it should be noted that values for pain thresholds remain subjective. Also, most participants did not describe the stimulation procedure as painful, but rather severely uncomfortable.

Secondly, pressure algometry can be difficult to measure consistently in this setting. In our study, we did not perform pressure algometry to the index and middle finger since we found the measurements at this location to be less reproducible compared to the thenar musculature.

Thirdly, force production measurements are prone to error when additional muscle groups are used. Despite meticulously positioning the participant’s arm in a “neutral” position, one cannot fully prevent the possible contribution of more proximal muscle groups. A possible solution for further research on the muscle power of the median nerve could be the use of a validated handgrip-strength meter which was used in the trial by Serrano-Munoz et al.^18,19^.

Finally, contrary to Kim et al., the pressure algometer test was not performed to the skin at the tip of the index or middle finger. Prestudy testing revealed that the algometer slipped of the finger very easily with the increasing pressure applied. Therefore, the pressure algometer test was performed to the thenar side of the palm. Sensory testing at 2 different locations intuitively raises substantial systematic questions of comparability. However, it has to be emphasized that the sensory supply area of the median nerve covers both the thenar side of the palm and the skin over palmar side of the thumb, index and middle finger.

Based on the results of this study, tMFAEC appears to have limited applications within the field of clinical anesthesia. There remains a substantial difference between the Semmes–Weinstein Monofilament examination and a nociceptive pain stimulus (e.g. skin incision) during surgery. The pressure pain threshold did not gain a significant improvement with tMFAEC, indicating that surgery with tMFAEC would still be uncomfortable for the patient. Based on existing literature, a more promising future for tMFAEC could be seen in the field of medical rehabilitation. Studies have already described the significant effects of tMFAEC on muscle spasms in animal models and demonstrated that alternating current in the kiloHertz frequency was able to reduce muscle spasms, which is promising for patients who suffer from chronic painful muscle contractions^20–22^.

## Conclusion

This study demonstrates that tMFAEC successfully inhibits sensory nerve activity. The use of tMFAEC with both types of electrodes and stimulation frequencies causes a transient tactile sensory deficit.

Overall, better results were achieved using a 2 kHz stimulation frequency with a smaller electrode contact area when compared to 10 kHz stimulation.

Pressure algometry and motor inhibition were not significantly reduced by tMFAEC but showed a tendency towards effect in specific subgroups.

Future research should focus on optimal stimulation frequencies, the most appropriate electrode size, and the most effective anatomical landmarks for the application of tMFAEC electrodes. At present, the clinical applicability (i.e. a potential adjuvant treatment for a failed locoregional anesthetic blockade) is probably still limited due to limited clinical effectiveness and significant uncomfortable side-effects of tMFAEC.

## Supplementary Information

Supplementary Information.

## Data Availability

The datasets used and analysed during the current study are included in this published article.

## Abbreviations

IQR: Interquartile Range
MVC: Maximum Voluntary Contraction
NRS: Numeric Rating Scale
tMFAEC: Transcutaenous medium-frequency alternating electrical current
