# Supplementary material for: Transcutaneous electrical nerve inhibition using medium frequency alternating current
Source: Sci Rep. 2022 Sep 1;12:14911. doi: 10.1038/s41598-022-18974-3 (PMC9437086; doi:10.1038/s41598-022-18974-3)
Supplement: Supplementary file 1 — Supplementary Information. [file 41598_2022_18974_MOESM1_ESM.docx]

**SUPPLEMENTARY**

| **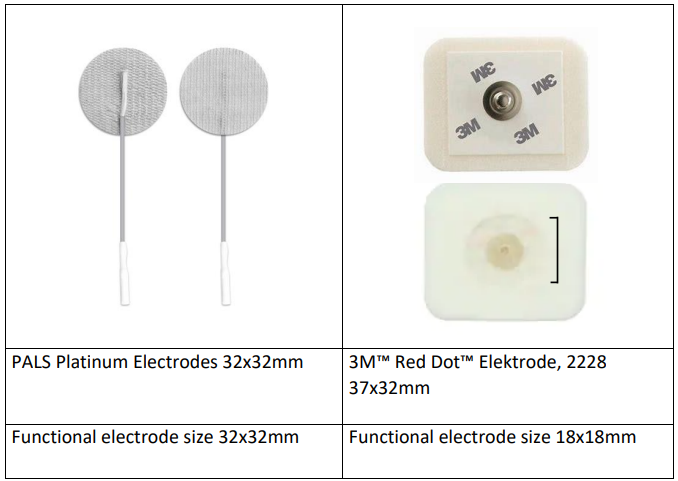** | |
| --- | --- |
| PALS Platinum Electrodes 32x32mm | 3M Red Dot Elektrode, 228 37x32mm |
| Functional Electrode Size 32x32mm | Functional electrode size 18x18mm |

**Fig. S1: Overview of the electrodes shape and size.** Functional electrode size is equal to the overall electrode size for the PALS Platinum Electrodes. On the contrary, functional electrode size is smaller than the overall electrode size for the 3M Red Dot Electrode.

| Table S1: NRS scores for the different stimulation conditions. NRS scores range from 0, indicating no pain, to 10, indicating the worst pain imaginable. Values for the NRS scores are presented as median (IQR). NRS = Numeric Rating Scale. | |
| --- | --- |
|  | NRS scores |
| PALS 10 | 1.00 (1.00 – 2.00) |
| PALS 2 | 2.00 (1.50 – 3.00) |
| 3M 10 | 2.00 (1.50 – 3.00) |
| 3M 2 | 2.00 (1.50 – 3.00) |

| Table S2: Tactile sensation, pressure pain, and MVC values. Values for the Semmes-Weinstein Monofilament test and force production test are represented as mean ± SEM. Force production values were normalized at baseline. Values for pressure pain threshold are shown as median (IQR). MVC = Maximum Voluntary Contraction. The virtual finger is defined as the sum of the index and middle finger force. | | | | | |
| --- | --- | --- | --- | --- | --- |
|  | Baseline | PALS 10 | PALS 2 | 3M 10 | 3M 2 |
| Log Tactile sensation (log(mg)) | 1.75 ± 0.09 | 2.14 ± 0.12 | 2.89 ± 0.22 | 2.38 ± 0.12 | 3.35 ± 0.25 |
| Tactile sensation (mg) | 56.86 ± 1.23 | 137.66 ± 1.32 | 779.83 ± 1.66 | 240.99 ± 1.31 | 2238.72 ± 1.77 |
| Pressure pain threshold (kg/cm^2^) | 6.32 (4.77-6.98) | 6.53 (5.03-7.30) | 6.46 (5.24-7.44) | 6.93 (5.66-8.66) | 7.20 (5.47-8.28) |
| MVC index finger (%) | 100.00 ± 0.00 | 117.32 ± 11.41 | 106.07 ± 10.48 | 103.08 ± 9.38 | 100.23 ± 11.04 |
| MVC middle finger (%) | 100.00 ± 0.00 | 99.90 ± 7.43 | 95.11 ± 7.77 | 91.6 ± 5.81 | 86.85 ± 7.10 |
| MVC virtual finger (%) | 100.00 ± 0.00 | 106.50 ± 8.34 | 98.57 ± 8.09 | 95.16 ± 6.51 | 90.82 ± 7.64 |
